# Using Video Technology and AI within Parkinson’s Disease Free-Living Fall Risk Assessment

**DOI:** 10.3390/s24154914

**Published:** 2024-07-29

**Authors:** Jason Moore, Yunus Celik, Samuel Stuart, Peter McMeekin, Richard Walker, Victoria Hetherington, Alan Godfrey

**Affiliations:** 1 Department of Computer and Information Sciences, Northumbria University, Newcastle upon Tyne NE1 8ST, UK; jason.moore@northumbria.ac.uk (J.M.);; 2 Department of Sport, Exercise and Rehabilitation, Northumbria University, Newcastle upon Tyne NE1 8ST, UK; 3 Department of Neurology, Oregon Health & Science University, Portland, OR 97239, USA; 4 Department of Nursing, Midwifery and Health, Northumbria University, Newcastle upon Tyne NE1 8ST, UK; 5 Northumbria Healthcare NHS Foundation Trust, Newcastle upon Tyne NE27 0QJ, UK; 6 Population Health Sciences Institute, Newcastle University, Newcastle upon Tyne NE2 4AX, UK; 7 Cumbria, Northumberland Tyne and Wear NHS Foundation Trust, Wolfson Research Centre, Campus for Ageing and Vitality, Newcastle upon Tyne NE4 9AS, UK

**Keywords:** wearable technology, inertial measurement units (IMUs), environmental context, artificial intelligence (AI), gait analysis, eye-tracking, privacy, ethics, thematic analysis

## Abstract

Falls are a major concern for people with Parkinson’s disease (PwPD), but accurately assessing real-world fall risk beyond the clinic is challenging. Contemporary technologies could enable the capture of objective and high-resolution data to better inform fall risk through measurement of everyday factors (e.g., obstacles) that contribute to falls. Wearable inertial measurement units (IMUs) capture objective high-resolution walking/gait data in all environments but are limited by not providing absolute clarity on contextual information (i.e., obstacles) that could greatly influence how gait is interpreted. Video-based data could compliment IMU-based data for a comprehensive free-living fall risk assessment. The objective of this study was twofold. First, pilot work was conducted to propose a novel artificial intelligence (AI) algorithm for use with wearable video-based eye-tracking glasses to compliment IMU gait data in order to better inform free-living fall risk in PwPD. The suggested approach (based on a fine-tuned You Only Look Once version 8 (YOLOv8) object detection algorithm) can accurately detect and contextualize objects (mAP50 = 0.81) in the environment while also providing insights into where the PwPD is looking, which could better inform fall risk. Second, we investigated the perceptions of PwPD via a focus group discussion regarding the adoption of video technologies and AI during their everyday lives to better inform their own fall risk. This second aspect of the study is important as, traditionally, there may be clinical and patient apprehension due to ethical and privacy concerns on the use of wearable cameras to capture real-world video. Thematic content analysis was used to analyse transcripts and develop core themes and categories. Here, PwPD agreed on ergonomically designed wearable video-based glasses as an optimal mode of video data capture, ensuring discreteness and negating any public stigma on the use of research-style equipment. PwPD also emphasized the need for control in AI-assisted data processing to uphold privacy, which could overcome concerns with the adoption of video to better inform IMU-based gait and free-living fall risk. Contemporary technologies (wearable video glasses and AI) can provide a holistic approach to fall risk that PwPD recognise as helpful and safe to use.

## 1. Introduction

Falls are common for people with Parkinson’s disease (PwPD), where impaired walking/gait is a leading contributing factor [1]. Fall risk may be further compounded when considering the context of free-living gait, where the nature of the surface or terrain on which walking is performed significantly impacts gait and stability [2]. This is mainly because gait adaptation strategies, which are crucial for maintaining stability, vary significantly with different walking surfaces [3]. This sensitivity to terrain is particularly relevant for older adults, including PwPD, due to deterioration in their sensory, motor, and cortical functions [4]. Typically, fall risk assessment includes subjective gait analysis in the clinic, but more recently, there has been a shift toward using digital technologies in free-living contexts to objectively understand impaired gait due to habitual behaviours [5]. Wearable inertial measurement units (IMUs), e.g., accelerometers and/or gyroscopes, are a contemporary and affordable approach enabling extended recording periods and quantification of clinically relevant gait characteristics, i.e., proxy intrinsic factors for clinicians to make informed fall risk assessments [6]. While IMUs can be used to differentiate soft and hard terrains [7], their effectiveness is compromised by individual differences in walking styles (including variations in stride length, speed, and foot placement). Moreover, IMUs alone do not provide crucial environmental data on context (i.e., extrinsic factors) that could be key to fully interpreting gait impairment and its contribution to fall risk [8]. Accordingly, current approaches for free-living gait analysis to understand fall risk in PwPD are limited.

Combining IMU data with environmental information to distinguish between intrinsic and extrinsic factors is crucial in order to progress to a better understanding of gait deficits in PwPD and arising fall risk [8]. Technologies like Global Positioning Systems (GPS) and tablets are not fit for purpose due to relying on outdated maps and self-reporting, respectively. Alternatively, static cameras have been suggested [9,10,11] with more contemporary approaches using wearable cameras [8,12], but these raise ethical/privacy concerns [12]. However, artificial intelligence (AI) has been suggested as a pragmatic and viable tool to uphold privacy during free-living video data capture, i.e., the use of AI-based computer vision to blur/obfuscate sensitive areas within the video frame [13]. In the referenced work, video data from wearable (eye-tracking based) video glasses display the environmental context to infer a better understanding of abnormal gait characteristics (e.g., high gait variability), which in turn provides better insight into fall risk. Yet, the approach used in the referenced study does not explore the additional functionality of the wearable video glasses used, i.e., eye-tracking [14].

Here, the purpose of the study is twofold. Firstly, we present a novel AI-based approach within a pilot study for eye-tracking to examine where the PwPD is looking using video-based glasses to inform fall risk from a clinician’s perspective while upholding privacy from data captured in the wild. It is proposed that the approach could be a key tool within free-living fall risk assessment to process video data and uphold privacy. Secondly, we showcase the video and AI-based pilot work to a group of PwPD and explore their perceptions regarding use of eye-tracking glasses and AI for fall risk assessment. We believe that this is an important aspect of this work, as several studies have highlighted the need to understand patient perceptions to identify potential barriers to the adoption of contemporary technologies [15,16,17,18,19,20,21,22,23,24,25]. This study is timely, as it investigates a potentially transformative approach with contemporary technologies to advance practice and to suggest testable techniques for future research and rehabilitation approaches in fall risk assessment. The work is strengthened by exploring the perspectives of PwPD.

## 2. Methods

A mixed-methods approach was undertaken to realise the breadth of this study. Firstly, a novel AI-based approach was presented in a pilot study to propose how the contextualisation of IMU gait with eye-tracking videos (to determine where the PwPD is looking) could be implemented while considering privacy features. Secondly, the proposed AI-based contextualisation to better inform gait within free-living fall risk assessment was discussed within a PwPD focus group. Participants were identified using purposive sampling techniques to ensure that the selection of PwPD had the relevant experience with undertaking free-living wearable-based gait research.

### 2.1. Participant Recruitment

The study was approved by the Northumbria University Institution review board (Ref: 44692, approval date: 12 April 2022). All participants gave written informed consent prior to enrolment (for the development of the AI-based model, focus group, and other data collection).

PwPD participants were recruited locally through networks within Northumbria University. People were excluded if they had significant cognitive impairment such that they could not understand instructions for focus group engagement and discussion. Inclusion criteria were as follows:

- Had received a clinical diagnosis of PD;
- Prior experience of participating in wearable-gait research;
- Familiar with technology, e.g., regular user of smartphones, tablets, applications [26];
- Willing to attend focus groups and consent to be audio-recorded;
- English-speaking and literate.

Additionally, 7 young adults (6M:1F, 23–32 years) were recruited to wear the video glasses only. The purpose here was to gather video data to develop the AI-based training dataset. Young adults were recruited through word of mouth and excluded if they had any functional impairments.

### 2.2. Video Data Collection

To acquire the required data, PwPD were recruited to wear an IMU and video eye-tracking glasses within a lab and free-living scenarios. Specifically, PwPD wore the McRoberts MoveMonitor IMU (The Hague, The Netherlands) mounted at the lower back (5th lumbar vertebrae, L5) and Pupil Labs (Berlin, Germany) Invisible eye-tracking glasses for approximately 2 h in a controlled lab, and then in their own homes and the surrounding community. In the lab, PwPD were asked to complete one 2-min walk at their usual pace to acquire a baseline for their gait characteristics on flat/level terrain. After, PwPD returned home, and while no scripted tasks were provided, participants were encouraged to ensure full traversal of their environments while wearing the technologies. Additionally, young adults wore the Pupil Labs Invisible eye-tracking glasses only for a period of 2 h walking a scripted route in Northumbria University and their own homes.

#### Datasets

For developing the AI model (Section 2.4), pre-trained weights from the Microsoft Common Objects in Context (MS COCO) dataset [27] were first initialised before fine-tuning on a new (local) dataset (with a pragmatic 80:20 split ratio) with the final classification layer of the model adapted to match our given classes (Table 1). Specifically for fine-tuning, 10 h of local video data were obtained, with >1500 frames manually extracted for annotation and used in training the model. The acquired dataset was then annotated utilising the LabelImg [28] python tool with selection of 4 categories and 18 classes (Table 1) which the research team deemed pertinent to fall risk or privacy. A total of 1542 frames were selected and annotated, and these included a broad spectrum of environments gathered from:

1. 7 young adults: The scripted route consisted of 2 loops of 10 interior environments followed by a walk around 10 different exterior environments to provide rich and diverse scenarios critical for training and validation.
2. 3 PwPD: 240 frames were manually extracted from the total number accumulated from each PwPD while ensuring unseen data remained for testing.
3. To bolster the variety of the collected environments, 4 further videos (approx. 240 mins) were downloaded from video-sharing websites (CC-BY licence) of first-person-view exterior environments and annotated as previously described.

### 2.3. IMU Gait Data Collection

IMU data collected during all walks were processed via a validated segmentation algorithm which identified walking/gait from other activities [31]. Subsequently, initial contact (IC) and final contact (FC) events within the gait cycle were quantified using a validated algorithm specifically designed for a wearable located on L5 [32]. Arising IC and FC events helped to estimate temporal gait characteristics. Here, the mean, standard deviation (STD), and asymmetry (Asy.) of step, stance, and swing times were used for exploratory purposes only to highlight the benefits of the proposed AI.

### 2.4. Using AI: Context and Privacy

For the purpose of this pilot study, a Yolov8 based object detection algorithm [33] was proposed and fine-tuned on a novel dataset captured across free-living environments (Section 2.4.1). To fulfil the purpose of providing automated environmental context to IMU gait data, additional features were developed to provide a naïve assumption of the participants’ walking paths (Section 2.4.2), and a methodology was developed for detecting overlap (Section 2.4.3) between both walking path and the participants’ gaze locations.

#### 2.4.1. Object Detection Algorithm

A fine-tuned YOLOv8 object detection algorithm was used as the baseline for the object detection algorithm. The YOLOv8 network is a state-of-the-art deep learning model that can detect objects in real time with high accuracy and speed, and is provided by the *ultralytics* library [33]. The model was trained using the Distribution Focal Loss (DFL) loss function.

#### 2.4.2. Walking Path

**Figure 1:**
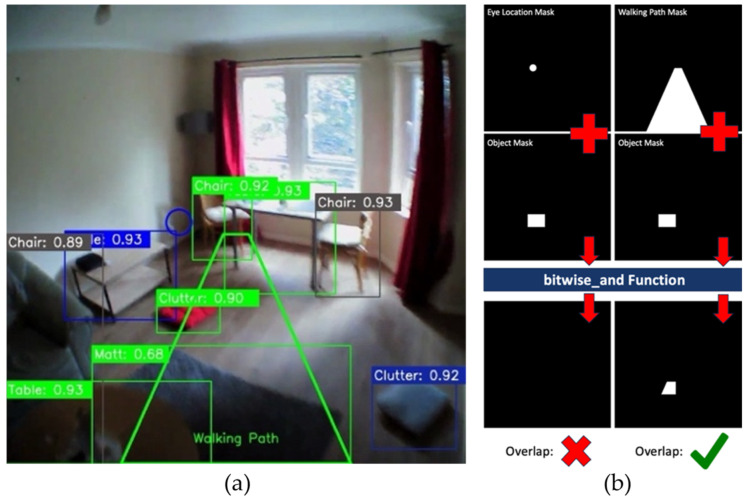
(**a**) Example output from system with rendered walking path—some bounding boxes overlap but that is typical during object detection in a busy environment as the example here is to illustrate what the AI detects e.g., chairs (grey boxes) and (**b**) example application of the overlap detection for eye location (blue outline circle on left in (**a**) and white circle on right) and walking path.

A naïve walking path was also incorporated into the newly developed model (Figure 1). This was achieved by specifying the point coordinates of a trapezoidal/perspective-warped rectangle shape (Figure 1a) to encompass the assumed walking path of the participant to provide further context for possible gait fluctuations. The width of the trapezoid at the bottom (base) of the frame was set to the middle 50% of the frame to capture a logical and wide area directly in front of the participant. The width of the trapezoid at the top was narrower, set to a fraction of the frame width to represent the converging perspective of the participant’s forward view. This becomes necessary when dealing with examples such as raised pathways and stairs. For example, although a raised path may be in frame, unless it is within the immediate walking path, it would not explain potential abnormalities in a participant’s gait.

#### 2.4.3. Overlap Detections

At the core of the model is the detection of overlap between (i) potential hazards (obstacles), (ii) eye location (i.e., where the person is looking), and (iii) immediate walking path. Binary segmentation masks are generated (Figure 1b) using the bounding box coordinates produced from the object detection algorithm for each object. This same process is repeated for eye location and walking path, allowing for detection of overlaps by performing a *binary_and* operation with the resulting binary mask containing pixels with a value of 1 where overlap occurs (Algorithm 1). This process, however, incurs computational overhead, particularly in scenarios with numerous objects or high-resolution images, potentially leading to a notable decrease in frame rate. Furthermore, the complexity increases linearly with the number of objects present in the scene. To mitigate the effect of this, a high degree of overlap accuracy can be retained with significantly reduced overhead by downscaling the masks to a resolution of 200 × 200 px.
**Algorithm 1:** Algorithm for Detecting Overlaps**Require:** List of detected objects and co-ordinates**Ensure:** Boolean return value of whether overlaps1.   eye_mask = zeros(frame_width, frame_height, 1, uint8)2.   obj_mask = zeros(frame_width, frame_height, 1, uint8)3.   path_mask = zeros(frame_width, frame_height, 1, uint8)4.   **fill** path_mask with 1 at path_location5.   **fill** obj_mask with 1 at obj_location6.   **fill** eye_mask with 1 at eye location7.   eye_overlap = bitwise_and(eye_mask, obj_mask)8.   path_overlap = bitwise_and(path_mask, obj_mask)9.   path_overlap = bool(path_overlap. unique > 1)10. eye_overlap = bool(eye_overlap.unique > 1)11. **return** eye_overlap, path_overlap

### 2.5. Privacy Features

**Figure 2:**
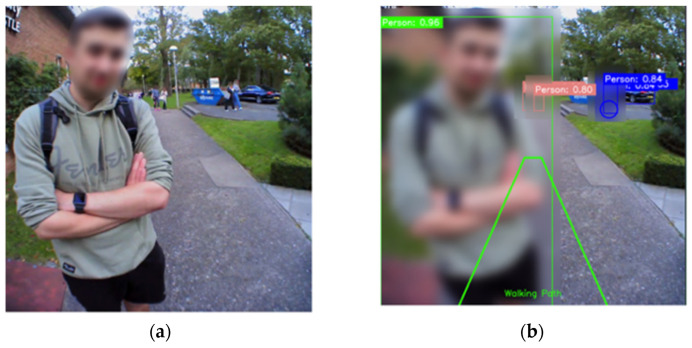
Example input and output from the system with privacy features enabled. (**a**) Depicts the raw input to the proposed model (here the face has been manually anonymised for privacy), and (**b**) output from the model with AI person-based anonymisation (foreground: green box with blur), walking path (green trapezoid) and obstacle detection (background).

To generate privacy-conscious videos, we defined multiple classes as sensitive (Table 1) [13]. For example, when detected, the bounding box coordinates were used to overlay a Gaussian blur obscuring any of the privacy-based detections (Table 1 (4), Figure 2) (Algorithm 2). This ensured that the privacy of the participants was protected while still providing valuable insights into their gait patterns and fall risk in free-living environments.
**Algorithm 2:** Algorithm for Selective Blurring**Require:** List of detected privacy objects and co-ordinates**Ensure:** Blurred Frame1.  ROI = frame**[x1:y1, x2:y2]**2.  blurred_roi = gaussian_blur(ROI, (157, 157))3.  frame**[x1:y1, x2:y2]** = blurred_roi4.  **return** frame

### 2.6. Focus Group: Participant Recruitment

A mini focus group and thematic analysis study design were used to gain insight into PwPD perceptions and concerns on the use of the technologies and novel applications proposed in Section 2.4 and Section 2.5. Here, the use of a focus group enabled the generation of ideas through interaction to discuss thoughts, opinions, attitudes, and perceptions. Moreover, the mini focus group approach was chosen a priori due to the plausible situation where only a small potential pool of participants could be included [34], given the nuanced topic. Accordingly, recruitment was halted once the minimum suggested threshold for a mini focus group was reached (i.e., n = 4) [35]. The standards for reporting qualitative research (SRQR) were adopted for this study [36], as shown in the Supplementary Material.

#### Data Collection and Analysis

The focus group took place at the Coach Lane Campus, Northumbria University, Newcastle upon Tyne, and comprised four PwPD to enable all participants to make in-depth contributions [37]. Specifically, the focus group was designed for a small number of participants only, given the complex topic of contemporary technologies. An experienced focus group facilitator (AG) and another member of the research team (JM) were present. The focus group was recorded and then transcribed verbatim. Field notes were also taken.

**Figure 3:**
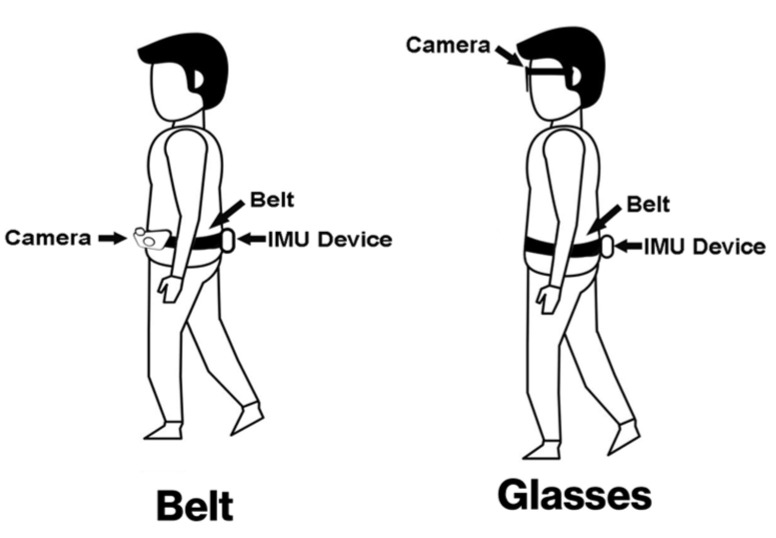
(**Left**): The literature generally describes wearable cameras on the torso (e.g., belt attachment at the waist). (**Right**): Focus group participants were shown an alternative and more contemporary video data capture modality: wearable video glasses. Both approaches were used to add context to IMU-based walking/gait in fall risk assessment.

**Figure 4:**
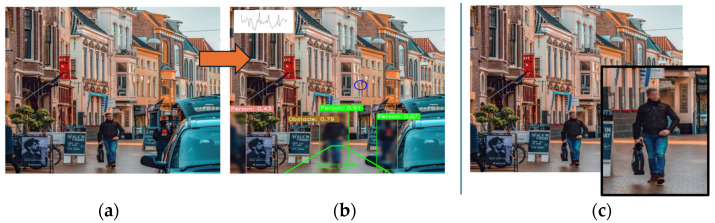
(**a**) A proposed original (i.e., not augmented) image for input into the proposed AI approach; (**b**) the output of the AI with applied anonymisation techniques, obscuring the whole person within the image (green box with blur), and identification of obstacles (other boxes) in the walking path (green trapezoid) and finally, IMU data is overlaid as an example. (**c**) Displays the example output of a current best-in-class (e.g., Google Maps) anonymisation when applied to the same image (where only the face is blurred).

The focus group started with PwPD being shown a short demonstration on IMU-based walking/gait with (Figure 3 and Figure 4) the inclusion of topics (i) and (ii), as shown below. The focus group audio recording was transcribed verbatim (JM) and validated by another researcher (AG).

i. Wearable cameras from the literature [8,12], first via a belt (GoPro https://gopro.com) and secondly via glasses (Pupil Labs https://pupil-labs.com) (Figure 3).
ii. The proposed AI (developed here) was used to analyse video data to contextualise and uphold privacy (Figure 4).

A semi-structured format was used whereby participants were encouraged to digress and fully explain new ideas and thoughts. When all key issues had been fully discussed and probed and no additional issues had been raised, the facilitators summarised the perceptions and concerns expressed by the group. All data were anonymised, and participants were referred to numerically. Facilitators discussed the transcript, notes, and arising themes after the focus group and agreed that data saturation had occurred and that no further focus groups needed to convene [38].

## 3. Result

### 3.1. Video and AI: Informing Free-Living Fall Risk Assessment

By combining the stages of methodological assessment (i.e., object detection, walking path, overlap detection) a final model capable of providing a fully contextualised free-living gait assessment to better inform fall risk was produced. Within this section, we present the results of the proposed approach. Later, we present the results of the focus group, detailing the perspectives of PwPD on the use of the approaches proposed in this paper.

#### 3.1.1. Object Detection Algorithm

Using the collected dataset, the YOLOv8 algorithm was trained for a course of 100 epochs converging at epoch 69 within a timeframe of 4 h. The models achieved a best validation mAP50 of 0.81 at epoch 69, showcasing the potential of this algorithm within real-world deployment (Table 2). Inferencing of the model with the additional computational complexity of overlap detection allowed videos to be processed at a rate of 21 frames per second (fps), achieving near real-time performance. This was conducted on a Windows-based machine comprising an AMD Ryzen 3600 k CPU (Santa Clara, CA, USA), an Nvidia RTX 3070 (8 GB VRAM) GPU (Santa Clara, CA, USA), and 24 GB of RAM.

#### 3.1.2. Applying the Model

**Figure 5:**
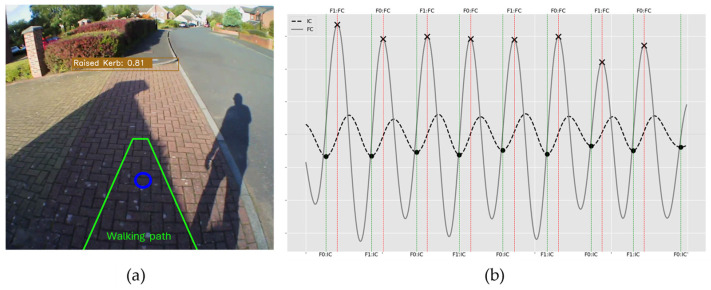
Free-living #1. (**a**) View from video eye-tracking glasses of a participant navigating a level and unobstructed terrain with superimposed outputs from the object detection model. (**b**) The corresponding IMU gait data during the same period of walking, with peaks and troughs of each signal to estimate initial contact (IC) and final contact (FC) times in the gait cycle. Gait signal (**b**) shows a stable and rhythmical gait.

To demonstrate the utility of the proposed approach, this section will use unseen data from a single participant only to convey the importance of classifying the environmental context (Figure 5). During an outdoor walk (defined here as Free-living #1), the proposed object detection model (Figure 5a) classified no potential obstacles (or fall risks) within the participant’s immediate walking path (green trapezoid). The participant’s attention was on their immediate path (blue circle overlapping trapezoid). This classification of a low fall risk from the object detection model was also reflected within the IMU signals and IC/FC events (Figure 5b), as the resulting plots displayed a consistent and stable signal, indicating a smooth gait without any abnormalities (i.e., high variability). Table 3 contrasts the Free-living (#1 and #2) to the normative temporal gait characteristics (for this participant in the lab), confirming that there were no significant anomalies for this person.

**Figure 6:**
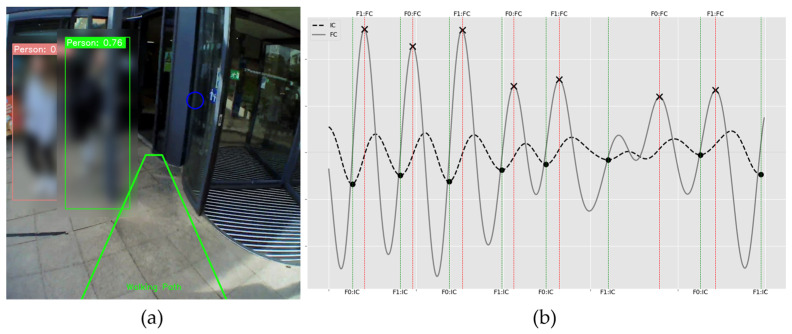
Free-living #2. (**a**) View of a participant navigating busy terrain (with a revolving door), with superimposed outputs from the object detection model shown (persons via blurred boxes, walking path via trapezoid and circle for via detection on revolving door. (**b**) The corresponding IMU gait data during the same period of walking, with peaks and troughs of each signal to estimate initial contact (IC) and final contact (FC) times in the gait cycle. Gait signal (**b**) shows a less stable gait, with clear breakdown of the rhythmical pattern in the middle of the plot.

Figure 6 shows a different outdoor walk (Free-living #2). Within the participant’s view (Figure 6a), they navigated a door, and the algorithm detected multiple people within their immediate walking path. Accordingly, the participant adjusted their gait to those environmental factors and observed the door beyond their immediate path (Figure 6a). Table 3 displays the resulting gait characteristics arising from the corresponding gait signals (Figure 6b). Compared to controlled lab conditions, walking in free-living environments showed increased asymmetry values across all temporal parameters. This difference was also apparent between Free-living #1 (walking on an uncrowded pavement) and Free-living #2 (walking on a more crowded walkway). In this instance, the researcher’s perception without context would be an elevated fall risk. Here, we observed that the participant seemed to be naturally reacting to the environment, so the fall risk may not have been high, but of course, the instability (Figure 6b and Table 3) could suggest that some risk may occur.

### 3.2. Focus Group

Here, we move beyond the quantitative results and examine the perceptions of PwPD regarding the approach. Within the focus group, PwPD were shown potential methods of attachment for a camera-based system to capture the data required for our proposed model.

#### 3.2.1. Theme 1: Usability

When discussing attachment methods, participants had initial enthusiasm for adopting worn belt technology into their daily routines. However, reservations emerged regarding potential inconvenience during prolonged use. Participant #1 succinctly pointed out, “*It’s just gonna* [going to] *be probably bulky*”. and #2 concurred, “*Yes, certain daily activities might become restricted*”. Participant #1 added that adapting to the device would require practice for donning it and taking it off. Those concerns were echoed among all participants, with #2 and #3 discussing the need for assistance when attaching the technology. Participant #4 further elaborated on how the necessity of constantly taking it on and off in a workplace setting could lead to creative ways of avoiding its use. Participant #2 agreed, noting that after wearing it continuously for more than a week, they might struggle.

In contrast, participants exhibited a more favourable attitude toward video glasses, perceiving them as less conspicuous and more socially acceptable, e.g., “*I’d go with the glasses*” and “*the glasses definitely seem to be the best option*”. Participants who wore prescription glasses were open to the idea of incorporating prescription lenses into the video glasses or wearing them over their regular eyeglasses. The ease of removing the glasses and putting them back on was a clear influence on participants acceptability of the video glasses. However, there was some discussion regarding the potential discomfort of wearing glasses, especially for those unaccustomed to them. For both technologies, all participants voiced concerns regarding functional practicalities (e.g., battery life and intricacies of charging).

#### 3.2.2. Theme 2: Fitting In

Participants were asked to share their perceptions of how others might react. Participant #1 suggested that devices “*seems like a talking point at* [a] *pub*”. Participant #2 anticipated that people would likely ask numerous questions. Participant #3 drew from previous experiences with other technologies: “*I’ve had some tech in my house and around the neighbourhood, and the neighbours were concerned. They asked about electrodes on my legs, and I did look rather funny*”. Participant #4 expected to draw attention from many people, speculating, “*I’ll probably be stopped by a lot of folks, wondering what all this is about*”.

The participants acknowledged that glasses might attract less attention than belt-mounted cameras, but could still raise public curiosity, leading people to inquire about their purpose. Participants emphasized the advantage of having control over when to wear or remove the glasses and expressed their intention to avoid wearing them in situations where privacy concerns might arise. Participant #5 exemplified this by stating, “*I would need to consider this when I’m teaching face-to-face as well*”.

#### 3.2.3. Theme 3: Data Capture and AI Processes

Participants highlighted that audio data arising from cameras should be disabled or deleted, as they deemed it irrelevant for fall risk assessment. Participants noted the value of AI as a tool to uphold privacy, suggesting that AI should blur/remove sensitive information, e.g., faces, documents, screens, or sensitive areas (e.g., bathrooms), from videos. However, participant #4 noted the need to check or correct errors stemming from any AI, “*especially as when you get to the point of the researchers actually viewing the processed video. I guess if I notice anything was left in. At that point they could go back and have that removed. So, I guess you’ve got checks in there.”*

Participants were interested in AI’s accuracy in terms of detecting sensitive material and anonymizing videos. They asked about the algorithms, the technology, and how it could handle different scenarios and environments. Accordingly, participant #2 preferred the scenario where the AI was the only thing that saw the raw video data and deleted it after processing. Interestingly, on the topic of daily video capture and AI, participant #1 drew a comparison to a common platform, saying: “*You know if that was Google earth, you’d see the body and just the face blurred and that’s acceptable. Everybody that uses google earth has accepted and I think this this is probably a safer option*”.

## 4. Discussion

To the authors’ knowledge, this is the first study to propose a method to contextualise IMU gait in order to better inform free-living fall risk in PwPD while using eye-tracking to examine where the person is looking while preserving privacy. Moreover, we also believe that this is the first focus group-based study to investigate users’ (i.e., PwPD) perceptions of wearable camera technology and AI to inform free-living fall risk assessment.

Falls tend to occur where people spend the most time (e.g., at home, in gardens, on walkways), so studies are needed to identify where falls occur in free-living environments as well as the various potential hazards. While most prior studies on fall risk have focused on analysing spatial, temporal [39], and turning gait characteristics [40] extracted from wearable IMUs in free-living contexts, it is critical to emphasize the sensitivity of these parameters to the walking environment and terrain [41]. Previous studies have also shown that these differences might stem from the IMU’s attachment location as well as the specific algorithms used for detecting IC-FC moments [42,43]. Our findings further illustrate this point, revealing that variations in the mean and asymmetry of temporal parameters across two distinct free-living environments do not inherently indicate a heightened fall risk. This observation underscores the importance of considering the environmental context when interpreting gait parameters for fall risk assessment, suggesting a nuanced approach that accounts for the specific characteristics of different walking scenarios.

Findings and insights from this study could have pragmatic implications for contemporary fall risk assessment research beyond the clinic, harnessing contextual and automated methods. For example, the approach is data-driven for personalised fall risk assessments, which could enable home modification to reduce fall risks. Furthermore, the focus group’s discussions regarding wearable cameras illuminated critical considerations for their integration into a fall risk assessment system. Participants’ preferences for attachment methods, coupled with concerns about public perceptions and comfort, emphasize the need for a user-centric approach. A full system that incorporates wearable cameras must consider user comfort and discretion to ensure participant compliance and data quality. Research should focus on designing camera systems that strike a balance between capturing rich contextual information and minimizing intrusiveness. The introduction of wearable glasses as a less conspicuous alternative presents an opportunity for innovation in camera technology. Exploring the feasibility of incorporating these glasses into a full system, along with addressing concerns about battery life and logistics, could enhance the practicality and user acceptance of wearable cameras.

### 4.1. Proposed AI Model

We present one possible AI approach to improve free-living fall risk assessment via wearable eye-tracking glasses which provides envionmental context as well as information on where the PwPD is looking. The AI method can detect and contextualize objects and/or hazards in free-living environments (and if the PwPD looks at them), providing valuable insights into gait patterns and fall risks beyond the lab. We demonstrated the accuracy and robustness of the method (mAP50 0.81) on a new dataset of video data captured by eye-tracking glasses worn by PwPD during their daily activities.

**Figure 7:**
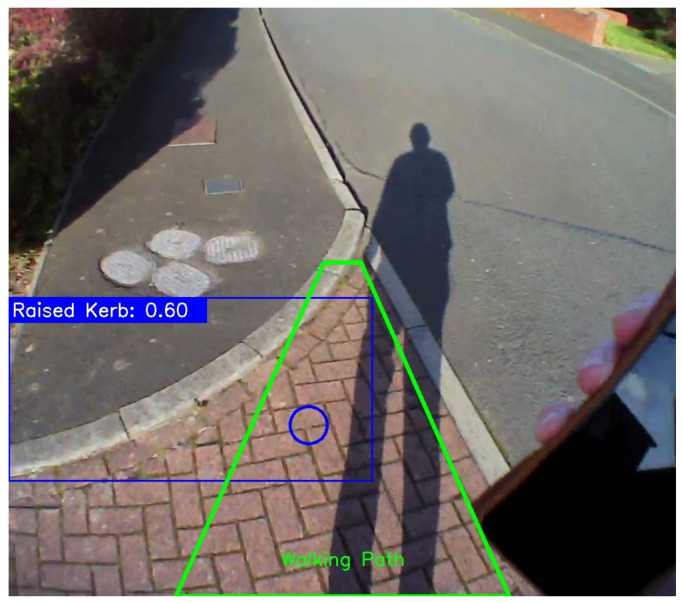
A later view from Free-living #1. Here, the eye-tracking (blue circle) was examined to understand whether the PwPD directly looked at the raised curb beyond or within their immediate path (green trapezoid), but they did not.

The proposed AI method has important implications for improving fall risk assessment in free-living environments. Current approaches to contextualising fall risk assessment methods are subjective or use technology that is not fit for the purpose, e.g., tables or maps, which do not capture the full complexity of gait and its interactions with environmental factors within real-world settings [44]. Using video-based eye tracking (and AI to automate analysis) could enable researchers to obtain a more accurate and holistic picture of gait impairments and fall risks for individual PwPD within their own settings, in addition to tailoring interventions accordingly. For example, Figure 7 shows a later view from Free-living #1 (i.e., Figure 6). In this instance, the video was watched by researchers to observe whether the PwPD examined (i.e., looked at) their upcoming or immediate path to identify the potential hazard of the raised curb, but they did not. Although no trip/stumble or fall was recorded and there is no comparison with clinical scores (e.g., Hoehn and Yahr Scale) in this study, the technology alludes to how gaze (viusal attention) in PD could be used to enhance our understanding of falls in free-living environments [45].

### 4.2. Perceptions

Here, we explored the perceptions and concerns of PwPD regarding the use of wearable cameras and AI for fall risk assessment and detection. We identified three main themes from the data analysis: usability, fitting in, and data capture and AI processes. Our findings provide valuable insights for the future development and implementation of comprehensive and objective fall risk assessment systems that incorporate wearable cameras and AI. Previous studies have mainly focused on the user perspectives on telemedicine, personal emergency systems, and statically mounted cameras [10,11,12,13,14,15,16,17,18,19,20]; however, none to date have begun to investigate the perspective of wearable cameras combined with other wearables (e.g., IMUs) or the ever-increasing use of AI integrated into their everyday lives for assessing fall risk. Our study addresses this gap by exploring the views and experiences of PwPD who have used wearable sensors for gait analysis and are familiar with the potential benefits and challenges of these technologies. Our study also complements and extends the findings of other qualitative studies that have investigated the attitudes and acceptance of older adults toward the use of wearable devices and cameras [19,20] for health monitoring and fall prevention. These studies have revealed various factors that influence the adoption and use of these technologies, such as perceived usefulness, ease of use, comfort, discretion, privacy, and control [20]. Our study confirms and elaborates on some of these factors, such as the preference for wearable glasses over belt-mounted cameras, the need for control over data capture and AI processing [19,20,32], and the importance of addressing privacy concerns [32]. Moreover, our study provides novel insights into the specific features and design aspects of wearable cameras and AI that PwPD would like to see in a fall risk assessment system, such as the ability to disable audio recording, blur sensitive information, and correct errors.

Our study has several implications for the development and implementation of wearable cameras and AI for fall risk assessment and detection in PwPD. First, it suggests that user-centric and participatory approaches are essential to ensure the usability, acceptability, and trustworthiness of these technologies. Second, it indicates that wearable cameras and AI should be integrated with other sensors, such as IMUs, to provide a holistic and comprehensive assessment of both the intrinsic and extrinsic factors that contribute to fall risk. Third, it highlights the need for transparent and ethical communication and education about the benefits and safeguards of these technologies, as well as the involvement of clinicians and caregivers in the decision-making and feedback processes. By addressing these implications, future research can advance the field of fall risk assessment in PwPD, improving safety and quality of life.

The focus group discussions also revealed participants’ interest and curiosity about the AI technology and its role in anonymizing video data. Participants asked various questions about the algorithms and the technology, as well as how it could handle different scenarios and environments. They also expressed their preference for having control over the AI processes and being able to check or correct errors. These findings suggest that participants were not only concerned about privacy, but also engaged and informed about the potential benefits and challenges of AI. This is consistent with previous studies that have found that older adults are willing to adopt new technologies if they perceive them as useful, safe, and easy to use [19,20]. Moreover, participants’ comparison of the proposed AI system to Google Earth indicates that they were familiar with existing commercial applications of AI and video technology, and that they had (some) expectations and standards for the quality and performance of the system. This highlights the importance of developing AI systems that are transparent, reliable, and user-friendly, and that can meet or exceed the users’ expectations.

The focus group provided valuable insights into the perceptions and concerns of PwPD regarding the use of wearable cameras and AI for fall risk assessment. The study also demonstrated the feasibility and acceptability of conducting a focus group on this topic, and the potential of using this method to elicit rich and nuanced data from PwPD. The study contributes to the literature by exploring a novel and innovative approach to enhance free-living fall risk assessment; suggests testable hypotheses for future research, such as the impact of wearable cameras and AI on the accuracy and comprehensiveness of fall risk assessment; and assesses the effect of user-controlled features and interfaces on the trust and acceptance of the system. The study thus informs and advances the research on and practice of using contemporary technologies for fall risk assessment in PwPD. Arising discussions on AI technology and its role in anonymizing video data further underscore the need for privacy and ethical considerations in any development. Research should focus on the development and validation of AI algorithms capable of automatically anonymizing video data while preserving data integrity and accuracy [33]. However, the participants flagged AI’s accuracy and its adaptability to various scenarios to highlight the importance of ongoing research and to refine, check, and improve these algorithms [4,21]. Interestingly, participants showed awareness of the use of existing (commercial) camera-based technology and AI in public spaces to facilitate a useful resource while upholding privacy. The introduction of technologies discussed here as tools within fall risk assessment could be considered a viable opportunity rather than an ethical threat. AI methods should be embraced in video-based fall research, as many contemporary commercial technologies showcase usefulness in other aspects of daily life. Participants’ desire for control over when to use wearable glasses underscores the need for user autonomy. Research should explore user-controlled features that empower individuals to manage data capturing. Furthermore, participant discussion and questions about error correction mechanisms highlight the importance of developing user-friendly interfaces that allow individuals to monitor and adjust the AI’s performance. This user-centric approach can enhance user trust and acceptance of the technology.

### 4.3. Privacy

Focus on the regular use and integration of video technologies primarily relates to privacy [24,25]. People often express reservations about the intrusion into their private lives and the collection of sensitive data [25] arising from cameras [24,25]. However, the latter is imperative to recognize the absolute context relating to falls, and AI can appropriately uphold/protect privacy by analysing the data and/or obfuscating sensitive parts of a video/image [12,46,47]. For example, recent advancements in machine learning algorithms have shown promise in addressing privacy concerns by enabling selective anonymisation techniques and approaches [48,49,50], making people more comfortable with the incorporation of these technologies into their everyday lives [24,25]. Integrating AI into fall risk assessment tools could enhance the accuracy and objectivity of the analysis, providing clinicians with better insights into intrinsic and extrinsic factors [8]. However, it is crucial to approach the integration of AI with sensitivity to PwPD’s perceptions, ensuring transparent communication about the benefits and safeguards in place to address privacy concerns.

## 5. Limitations

Only a small number of PwPD were recruited, and a limited dataset was curated as part of the pilot study. However, given the novel nature of the work, the numbers and data were sufficient to demonstrate the use of the approaches suggested in this paper. Ongoing work is curating more original data to improve accuracy and conduct a clinical study.

The focus group methodology used in this study has some limitations that should be acknowledged. The number of participants was small, and they were recruited based on their prior experience of participating in wearable gait research and their familiarity with technology. Moreover, the focus group participants may have been biased toward the acceptance of technology as they were recruited by purposive sampling to have a good understanding and/or appreciation of commercial technology. The exclusion of less technology-savvy participants may have altered the outcome of the focus group, particularly in relation to overall positivity regarding the adoption of technology. Here, the work is exploratory and may have benefited from (a larger number of) heterogenous participants [51].

## 6. Strengths

Despite the modest original datasets, a good mAP50 (i.e., >0.8) was achieved to suggest that our approach is powerful and able to categorically inform extrinsic factors in free-living fall risk. Ongoing work is curating more original data via PwPD in the northeast of England. Moreover, we showcase the power of our AI methods to provide an ethical approach for added contextual information, as well as to determine where a PwPD is looking using eye-tracking glasses (i.e., eye coordinates intersecting with their walking path and extrinsic objects). We believe this to be a first in free-living fall risk assessment, and that it will pave the way for how PwPD navigate their environment while trying to understand their visual attention/gaze behaviour [45,52].

Although a mini-focus group methodology was adopted, it facilitated a homogeneous group which evoked a rapport [53], i.e., participants with similar experiences were able to share insights and perceptions, which was important in the context of the case study. Although many (small) focus groups could be conducted, there is often a pragmatic realisation that data saturation occurs and a reduced number is sufficient [54]. The homogeneous and miniature approach allowed for a deeper exploration of contemporary experiences that gave rise to an insightful observation (i.e., comparison to Google Earth technology) that may not have been realised in a larger and less technology-aware group.

## 7. Conclusions

Video eye-tracking and AI-based computer vision can be used to contextualise IMU-based gait to comprehensively inform free-living fall risk assessment (i.e., extrinsic objects and/or hazards and whether the PwPD looks at them). The model proposed here operates in near-real time and includes a means to uphold privacy. Perspectives from PwPD offer valuable guidance for the future deployment of the proposed contemporary approach to comprehensively quantifying fall risk assessment beyond the lab. The use of video and AI may hold potential to enhance the lives of PwPD, helping to better identify elevated fall risk at home and in the wider community. Specifically, wearable video-based glasses could be a useful tool to quantify extrinsic factors for fall risk assessment (better informing intrinsic gait characteristics) while understanding where the users are looking during walks. Of course, practical issues like battery life and comfort (for all) need consideration, but video glasses and AI seem optimal as a contemporary approach.

## Figures and Tables

**Table 1:** Object Detection Classes**.**

Number	Category	Class	Rationale
1	Context	Stairs	Context to higher variability and asymmetry measures [8]
	Context	Doorway	Although FoG is not investigated here, it was deemed important to include doorwas as they can provoke FoG in (some) PwPD [29].
	Context	Shower	Context to the type of room
	Context	Sink	Context to the type of room
	Context	Toilet	Context to the type of room
	Context	Table	Context to higher gait variability and asymmetry gait characteristics (but can also provoke FoG in PwPD [30])
	Context	Bed	Context to the type of room
	Context	Signage	Fluctuations in gait signal may be due to participant pausing and reading signage
	Context	Vehicle	Context to the type of environment
2	Context/Fall risk	Chair	Potential tripping hazard due to obstruction
	Context/Fall risk	Animal	Potential tripping hazard with animals running between/in front of participant
	Context/Fall risk	Wet surface	Potential hazard due to slippery surface
3	Fall risk	Matt/rug/carpet	Potential tripping hazard due to change in surface friction and/or curled/folded edge
	Fall risk	Obstacle	Generic catch-all for potential obstructions
	Fall risk	Raised kerb	Tripping hazard
4	Context/privacy	Person	Detected person will be blurred, but also may evoke gait alteration due to navigating around that person
	Privacy	Screen	Detection of any screen (e.g., laptop/TV/phone) will be blurred
	Privacy	Book	Catch-all for any text-based document that will be blurred

**Table 2:** Object Detection Algorithm Training**.**

Epoch	Val Loss	mAP50
67	1.353	0.77
68	1.242	0.79
**69**	**1.124**	**0.81**
70	1.355	0.78

**Table 3:** Temporal characteristics from a single PwPD during 2 outdoor walks (Free-living #1 and #2) compared to the participants’ own normative values from a lab-based 2-min walk.

	Lab			Free-Living #1			Free-Living #2		
	Mean	STD	Asy.	Mean	STD	Asy.	Mean	STD	Asy.
Step (s)	0.55	0.01	0.01	0.52	0.01	0.02	0.66	0.20	0.09
Stance (s)	0.69	0.01	0.01	0.66	0.01	0.02	0.88	0.29	0.04
Swing (s)	0.41	0.01	0.01	0.39	0.01	0.01	0.46	0.06	0.09

## Data Availability

Video data cannot be shared due to privacy concerns. IMU data can be shared upon reasonable request by contacting the corresponding author.

## Supplementary Materials

The following supporting information can be downloaded at: https://www.mdpi.com/article/10.3390/s24154914/s1, Standards for reporting qualitative research (SRQR).
